# Trends in motives for trying to stop smoking: a population study in England, 2018–2023

**DOI:** 10.1136/bmjph-2023-000420

**Published:** 2024-03-22

**Authors:** Sarah E Jackson, Sharon Cox, Vera Buss, Jamie Brown

**Affiliations:** 1 Department of Behavioural Science and Health, UCL, London, UK; 2 SPECTRUM Consortium, Edinburgh, UK

**Keywords:** Public Health, Preventive Medicine, trends

## Abstract

**Introduction:**

Since 2020, people in England have lived through a global pandemic and national cost-of-living and healthcare crises, each of which might have affected motivations to stop smoking.

**Objective:**

To examine changes in the factors motivating people to stop smoking over this period.

**Methods:**

Data were drawn from a nationally representative monthly cross-sectional survey in England, 2018–2023. Participants were 5777 past-year smokers who made one or more serious attempt to quit in the past year. Participants reported factors contributing to their most recent attempt to quit. We estimated time trends in the proportion of attempts to quit that were motivated by (i) health concerns, (ii) cost, (iii) social factors and (iv) health professional advice, and calculated prevalence ratios (PRs) for the change in prevalence across the whole time series (May 2023 vs March 2018).

**Results:**

Up to 2020, one in two attempts to quit were motivated by health concerns (mean monthly proportion 51.0%), one in five by social factors (20.2%) and cost (19.9%) and one in six by health professional advice (16.5%). In 2020, the proportion of attempts to quit motivated by health concerns, social factors and cost increased—to high levels of 56.2%, 23.9% and 25.8%, respectively—and those motivated by health professional advice fell to 8.0%. Rises in health-related and social motives soon returned to baseline levels (52.0% in May 2023 vs 52.5% in March 2018; PR=0.99, 95% CI 0.86 to 1.14) or below baseline (16.0% vs 21.6%; PR=0.74, 95% CI 0.54 to 1.01), respectively. However, attempts to quit motivated by cost increased further during 2022–2023 (reaching 25.4% in May 2023 vs 19.1% in March 2019; PR=1.33, 95% CI 1.01 to 1.76) and those motivated by health professional advice remained suppressed (8.5% vs 14.2%; PR=0.60, 95% CI 0.40 to 0.89).

**Conclusions:**

Health concerns are the most common motive for trying to stop smoking. The relative importance of other motives has shifted since 2020, with cost motivating a greater proportion of attempts to quit, and social factors and health professional advice motivating a smaller proportion.

**WHAT IS ALREADY KNOWN ON THIS TOPIC**

**WHAT THIS STUDY ADDS**

- Among adults in England, health concerns have consistently been the most common motive for trying to stop smoking.
- The relative importance of other motives has shifted since 2020, with cost motivating a greater proportion of attempts to quit, and social factors and health professional advice motivating a smaller proportion.

**HOW THIS STUDY MIGHT AFFECT RESEARCH, PRACTICE OR POLICY**

- As cost is an increasingly important motive for trying to stop smoking, communicating the potential savings people can make by stopping smoking could be effective for motivating attempts to quit.
- Further research is required to establish why attempts to quit motivated by health professional advice remain suppressed and identify strategies to improve this.

## Introduction

 Health concerns are generally the primary motive for people trying to stop smoking.^1^ Social (eg, family pressure, effects on others, a friend or family member wanting to quit) and financial concerns and advice from a health professional are also commonly cited reasons for wanting to quit.^1^ However, since 2020, England has undergone a period of substantial societal instability, which might have caused people’s motives for trying to stop smoking to change. Understanding the factors motivating people to stop smoking, how they differ between key population groups and how this is changing over time is important for informing interventions to encourage and aid smoking cessation.^2^

From early 2020, the COVID-19 pandemic introduced a new health risk, which for people living in many countries around the world resulted in prolonged periods of reduced social contact and for some, isolation, and left many people experiencing financial strain.^3^

In England specifically, there has also been a series of economic and monetary policy changes that have led to a period of financial instability and stress. Since late 2021, there has been a cost-of-living crisis in the UK, whereby high rates of inflation have caused the cost of everyday essentials, like groceries, energy and household bills, to rise faster than average household incomes.^4^ Inconsistent government leadership and subsequent financial budgets also created a shock in financial markets, which saw the value of the pound fall and the cost of government borrowing and mortgage interest rates rise sharply.^5^ In addition, an ongoing healthcare crisis has seen increased pressures on the NHS and across the healthcare and social care sector, resulting in substantial delays for patients seeking emergency and primary care.^6^ It is possible that these pressures have increased the salience of the health risks associated with smoking and boosted people’s motivation (whether based on want or need) to stop smoking to save money. There has been a sustained rise in the proportion of smokers making attempts to quit since the start of the pandemic, which might have prompted others to also try to quit.^7^ It is also possible that delays and difficulties in accessing healthcare might have reduced the proportion of attempts to quit that are motivated by advice from a general practitioner (GP) or other health professional.

The impacts of the pandemic and other recent events have not been experienced equally across the population. For example, older adults were at higher risk of becoming seriously ill or dying from COVID-19,^8^ while younger adults experienced greater social and financial impacts,^9 10^ and women disproportionately carried the burden of additional care.^11 12^ The cost-of-living crisis has seen particularly high rates of food insecurity in households with children and those receiving state benefits.^13^ More recently, the healthcare crisis is likely to disproportionately affect groups who seek emergency care more frequently, including older adults and people from deprived areas.^14^ As a result, any increases in wanting to stop smoking for health or financial reasons might have been greater among these population subgroups. There have also been dramatic recent shifts in the prevalence of vaping in England and harm perceptions,^15^,^17^ and given the majority of people who vape are also current or recent ex-smokers, there might have been important changes in motives to quit smoking as a function of vaping status.

This study aimed to estimate time trends in motives for trying to stop smoking between 2018 and 2023 and explore differences by key potential moderators. Specifically, we aimed to address the following research questions:

1. How have the proportions of attempts to stop smoking that are motivated by (a) health concerns, (b) cost, (c) social factors and (d) health professional advice changed over the past 5 years?
2. To what extent have changes in motives for attempts to stop smoking differed by age, gender, socioeconomic position (indexed by occupational social grade), presence of children in the household and vaping status?

## Materials and methods

### Pre-registration

The study protocol and analysis plan were pre-registered on Open Science Framework (https://osf.io/cgy57/). We made one amendment, which was to reduce the number of age groups for tests of interactions, as we encountered problems with the model convergence.

### Design

Data were drawn from the ongoing Smoking Toolkit Study, a monthly cross-sectional survey of a representative sample of adults in England.^18^ The study uses a hybrid of random probability and simple quota sampling to select a new sample of approximately 1700 adults each month. Comparisons with sales data and other national surveys indicate that key variables, including sociodemographics, smoking prevalence and cigarette consumption, are nationally representative.^18 19^

Ethical approval for the Smoking Toolkit Study was granted originally by the UCL ethics committee (ID 0498/001). The data are not collected by UCL and are anonymised when received by UCL.

We used data collected from March 2018 (2 years before the start of the COVID-19 pandemic) through May 2023 (the most recent data at the time of analysis).

Data were initially collected through face-to-face computer-assisted interviews. However, social distancing restrictions under the COVID-19 pandemic meant no data were collected in March 2020 and data from April 2020 onwards were collected via telephone. The telephone-based data collection used a similar combination of random location and quota sampling and weighting approach as the face-to-face interviews, and comparisons of the two data collection modalities indicate good comparability.^20^,^22^

We limited our sample to past-year smokers (ie, current smokers or those who quit in the past year) aged ≥18 years who reported having made at least one serious attempt to stop smoking in the past year.

### Measures

#### Sample selection

Smoking status was assessed with the question: ‘Which of the following best applies to you?

- I smoke cigarettes (including hand-rolled) every day;
- I smoke cigarettes (including hand-rolled), but not every day;
- I do not smoke cigarettes at all, but I do smoke tobacco of some kind (eg, pipe, cigar or shisha);
- I have stopped smoking completely in the last year;
- I stopped smoking completely more than a year ago;
- I have never been a smoker (ie, smoked for a year or more).’

Responses *a* to *d* were classified as past-year smokers. Those responding *e* or *f* were excluded from the sample.

Past-year attempts to quit were assessed with the question: ‘How many serious attempts to stop smoking have you made in the last 12 months? By serious attempt I mean you decided that you would try to make sure you never smoked again. Please include any attempt that you are currently making and please include any successful attempt made within the last year’. Our sample was restricted to past-year smokers who reported making one or more serious attempt to quit in the past year.

#### Outcome variables

Motives for the most recent attempt to quit were assessed with the question: ‘Which of the following do you think contributed to you making the most recent attempt to quit?’

- Advice from a GP/health professional
- TV advert for a nicotine replacement product
- Government TV/radio/press advert
- Hearing about a new stop smoking treatment
- A decision that smoking was too expensive
- Being faced with smoking restrictions
- I knew someone else who was stopping
- Seeing a health warning on a cigarette packet
- Being contacted by my local NHS Stop Smoking Services
- Health problems I had at the time
- A concern about future health problems
- Attending a local stop smoking activity or event
- Something said by family/friends/children
- A significant birthday
- Pregnancy
- Just decided to quit
- The coronavirus outbreak (from April 2020 onwards)
- Other (please specify)

We reported descriptive data on each motive, aggregated across the study period. Time trends were explored for the following motives, which have been identified in previous studies as the most popular reasons for trying to stop smoking^123^,^25^:

- Health concerns (responses *j*, *k, o, q*)
- Cost (*e*)
- Social factors (*g*, *m*)
- Health professional advice (*a*)

We also included any relevant responses given by those who responded *r* (other reasons) under these categories (eg, general health reasons or concern about the effect on others).^23^ In a sensitivity analysis, we analysed current health problems (*j*) and future health concerns (*k*) separately to explore any differences.

#### Potential moderators

Age was categorised as 18–24, 25–34, 35–49, 50–64 or ≥65 years.

Gender was identified as man, woman or in another way and summarised descriptively. Those who identified in another way were excluded from the trend analyses by gender owing to low numbers.

Occupational social grade was categorised as:

- AB (higher and intermediate managerial, administrative and professional);
- C1 (supervisory, clerical and junior managerial, administrative and professional);
- C2 (skilled manual workers);
- D (semiskilled and unskilled manual workers);
- E (state pensioners, casual and lowest-grade workers, unemployed with state benefits only).

Presence of children in the household was categorised as 0 or ≥1.

Vaping status was assessed with series of questions that asked participants whether they were using an e-cigarette or vaping device to help them stop smoking, cut down the amount smoked, in situations when smoking is not permitted or for any other reason at all. Those who reported e-cigarette use in response to any of these questions were considered current vapers.

### Statistical analysis

Data were analysed using R v.4.2.1. The Smoking Toolkit Study uses raking to weight the sample to match the population in England on the dimensions of age, social grade, region, housing tenure, ethnicity and working status within gender. This profile is determined each month by combining data from the 2021 UK Census, the Office for National Statistics mid-year estimates and the annual National Readership Survey.^18^ The following analyses used weighted data. Missing data were removed on a per-analysis basis.

We reported descriptive data on the proportion of participants, aggregated across all survey waves, who endorsed each motive for trying to stop smoking and the proportion reporting key motives (health concerns, cost, social factors and health professional advice) by age, gender, social grade, children in the household and vaping status.

We used log-binomial regression to test the association of key motives for trying to stop smoking with survey month. Survey month was modelled using restricted cubic splines with five knots, to allow relationships with time to be flexible and non-linear, while avoiding categorisation.

To explore moderation by age, gender, social grade, children in the household and vaping status, we repeated the models including the interaction between the moderator of interest and survey month—thus allowing for time trends to differ across subgroups. Each of the interactions was tested in a separate model.

We used predicted estimates from our models to plot the prevalence of each outcome over the study period (overall and by moderating variables), alongside raw (weighted) data and reported prevalence ratios (PRs) for the change in prevalence across the whole time-series (May 2023 vs March 2018) alongside 95% confidence intervals (CIs) calculated using bootstrapping.

In a planned sensitivity analysis, we reran the analyses separately for current health problems and future health concerns.

### Patient and public involvement

The wider Smoking Toolkit Study is discussed several times a year with a diverse Patient and Public Involvement group, and the authors regularly attend and present at meetings at which patients and the public are included. Interaction and discussion at these events help to shape the broad research priorities and questions. There is also a mechanism for generalised input from the wider public: each month interviewers seek feedback on the questions from all 1700 respondents, who are representative of the English population. This feedback is limited and usually just relates to understanding of questions and item options. No patients or members of the public were involved in setting the research questions or the outcome measures, nor were they involved in the design and implementation of this specific study. There are no plans to involve patients in dissemination.

## Results

There was a total of 101 919 respondents to the survey between March 2018 and May 2023. Of the 17 812 who reported smoking in the past year, 17 031 (95.6%) provided data on past-year attempts to quit, among whom 5777 (33.9%) reported having made at least one serious attempt to quit in the past year and formed our sample for analysis.

### Overall estimates of motives for trying to stop smoking

Table 1 shows the proportion who reported each motive as contributing to their most recent attempt to quit, across the study period. Health concerns were most frequently reported (52.0%)—in particular, concerns about future health (35.5%). Cost was the next most frequent motive (22.7%), followed by social factors (19.0%) and health professional advice (11.6%). Other motives were endorsed by between 0.6% and 3.7% of participants.

**Table 1:** Proportion reporting each factor as a motive driving the most recent attempt to quit smoking

Motive*	% (95% CI)
Health concerns	
A concern about future health problems	35.5 (34.2 to 36.9)
Health problems I had at the time	19.0 (17.9 to 20.1)
The coronavirus outbreak†	6.3 (5.5 to 7.1)
Pregnancy	2.3 (1.8 to 2.7)
*Any health concern*	*52.0 (50.6 to53.4*)
Cost	
A decision that smoking was too expensive	22.7 (21.6 to 23.9)
Social factors	
Something said by family/friends/children	15.4 (14.3 to 16.4)
I knew someone else who was stopping	5.2 (4.6 to 5.8)
*Any social factor*	*19.0 (17.9 to20.1*)
Health professional advice	
Advice from a GP/health professional	11.6 (10.7 to 12.5)
Other reasons	
Seeing a health warning on a cigarette packet	3.7 (3.2 to 4.2)
Being faced with smoking restrictions	3.5 (3.0 to 4.0)
Just decided to quit	3.1 (2.6 to 3.6)
TV advert for a nicotine replacement product	1.3 (1.0 to 1.6)
A significant birthday	1.3 (1.0 to 1.6)
Government TV/radio/press advert	1.2 (0.9 to 1.5)
Hearing about a new stop smoking treatment	0.9 (0.6 to 1.1)
Being contacted by my local NHS Stop Smoking Services	0.9 (0.6 to 1.1)
Attending a local stop smoking activity or event	0.6 (0.4 to 0.8)
Don’t know/can’t remember	0.8 (0.5 to 1.0)
Not stated	7.0 (6.2 to 7.7)

Data are aggregated across the study period (March 2018 – May 2023; unweighted *n*n=5777) and weighted to match the population in England.

* Motives are not mutually exclusive, as participants were allowed to report multiple motives.

† Among participants surveyed from April 2020 onwards (unweighted *n*n=3792).

CI, confidence interval

Table 2 shows the proportion reporting the four most popular motives within participant subgroups, across the study period. While health concerns were consistently the most common motive for trying to stop smoking, reported by around half (range 47.6–55.9%) of participants in all subgroups, they were slightly more frequently reported by those aged over 35 and those without children in the household. Current health problems were more frequently reported as a motive by older (vs younger) adults, women (vs men) and those from less (vs more) advantaged social grades, while the opposite was observed for future health concerns. Those without children in the household were more frequently motivated by both current and future health concerns than those with children at home. Cost was more frequently reported as a motive by those from intermediate social grades (C1, C2 and D) and by those who reported current vaping. Social factors were more frequently reported by those who were younger, from mid-range social grades, had children in the household and reported current vaping. Health professional advice was reported more frequently by those who were older, women and from less advantaged social grades. The contrasting age gradients in the proportions of attempts to quit motivated by social factors and health professional advice meant that, of the two motives, health professional advice more frequently motivated attempts to quit among those aged over 55, while social factors more frequently motivated those aged 18–54.

**Table 2:** Proportion reporting key motives driving the most recent attempt to quit smoking by participant characteristics

		Motive*, % (95% CI)					
	*N* [Table-fn T2_FN4] †	Health concerns‡	Current health problems	Future health concerns	Cost	Social factors	Health professional advice
Age							
18–24	1190	49.5 (46.4 to 52.6)	13.1 (11.0 to 15.1)	35.1 (32.2 to 38.1)	24.8 (22.1 to 27.6)	22.6 (19.9 to 25.2)	6.2 (4.7–7.7)
25–34	1519	50.1 (47.3 to 52.8)	13.9 (12.1 to 15.8)	35.8 (33.2 to 38.4)	22.6 (20.3 to 24.9)	20.9 (18.7 to 23.2)	9.4 (7.8–11.0)
35–44	955	54.4 (51.0 to 57.8)	17.0 (14.5 to 19.6)	40.2 (36.9 to 43.6)	21.0 (18.2 to 23.7)	18.7 (16.1 to 21.4)	11.5 (9.3–13.7)
45–54	868	52.2 (48.7 to 55.8)	22.5 (19.5 to 25.5)	35.1 (31.7 to 38.4)	22.1 (19.1 to 25.0)	18.6 (15.9 to 21.4)	14.2 (11.7–16.7)
55–64	675	55.2 (51.1 to 59.3)	29.9 (26.2 to 33.7)	33.1 (29.3 to 36.9)	22.7 (19.3 to 26.1)	12.6 (10.0 to 15.2)	17.6 (14.4–20.8)
≥65	570	55.9 (51.5 to 60.3)	36.5 (32.2 to 40.8)	29.0 (25.0 to 33.0)	23.2 (19.4 to 26.9)	12.3 (9.3 to 15.3)	21.5 (17.7–25.2)
Gender§							
Man	2931	51.2 (49.2 to 53.2)	16.6 (15.2 to 18.1)	37.9 (36.0 to 39.9)	23.1 (21.4 to 24.7)	20.2 (18.5 to 21.8)	10.1 (8.9–11.3)
Woman	2786	53.0 (51.0 to 55.0)	21.7 (20.1 to 23.3)	32.9 (31.0 to 34.7)	22.4 (20.8 to 24.1)	17.8 (16.3 to 19.3)	13.5 (12.1–14.9)
Other	53	55.4 (42.1 to 68.8)	17.2 (7.0 to 27.5)	36.3 (23.3 to 49.3)	18.9 (8.3 to 29.5)	17.0 (6.9 to 27.1)	3.8 (0.0–8.9)
Social grade							
AB (most advantaged)	861	54.7 (51.3 to 58.2)	15.0 (12.6 to 17.4)	41.0 (37.5 to 44.4)	19.0 (16.3 to 21.7)	16.0 (13.4 to 18.6)	9.7 (7.7–11.7)
C1	2150	54.5 (52.4 to 56.7)	17.5 (15.8 to 19.1)	39.0 (36.9 to 41.2)	24.2 (22.3 to 26.0)	19.0 (17.3 to 20.8)	8.7 (7.4–9.9)
C2	1125	50.5 (47.5 to 53.6)	17.2 (15.0 to 19.4)	35.1 (32.1 to 38.0)	23.4 (20.8 to 26.0)	21.8 (19.2 to 24.3)	12.1 (10.1–14.0)
D	830	47.6 (43.9 to 51.2)	18.7 (16.0 to 21.5)	30.7 (27.3 to 34.1)	25.0 (21.8 to 28.2)	20.0 (17.2 to 22.9)	13.2 (10.8–15.7)
E (least advantaged)	811	53.5 (49.8 to 57.1)	30.3 (27.0 to 33.6)	30.3 (27.0 to 33.6)	19.9 (17.0 to 22.7)	16.1 (13.4 to 18.8)	16.5 (13.8–19.1)
Children in the household							
0	3955	53.4 (51.7 to 55.1)	20.9 (19.6 to 22.3)	37.1 (35.5 to 38.8)	23.0 (21.5 to 24.4)	16.7 (15.4 to 18.0)	11.6 (10.5–12.6)
≥1	1822	49.2 (46.7 to 51.7)	15.1 (13.3 to 16.9)	32.3 (29.9 to 34.6)	22.3 (20.2 to 24.4)	23.7 (21.6 to 25.8)	11.8 (10.2–13.4)
Vaping status							
Non-vaping	3798	51.9 (50.2 to 53.7)	19.1 (17.8 to 20.5)	34.6 (33.0 to 36.3)	21.4 (19.9 to 22.8)	17.1 (15.7 to 18.4)	12.3 (11.1–13.4)
Current vaping	1979	52.2 (49.8 to 54.6)	18.7 (16.9 to 20.6)	37.2 (34.8 to 39.5)	25.4 (23.3 to 27.4)	22.7 (20.7 to 24.7)	10.4 (9.0–11.9)

Data are aggregated across the study period (March 2018 – May 2023) and weighted to match the population in England.

* Motives are not mutually exclusive, as participants were allowed to report multiple motives.

† Unweighted sample size.

‡ Current health problems, future health concerns, the Ccoronavirus outbreak, or or pregnancy.

§ There were 7seven missing cases for gender; valid percentages are presented.

CI, confidence interval;

#### Time trends in motives to stop smoking

**Figure 1:**
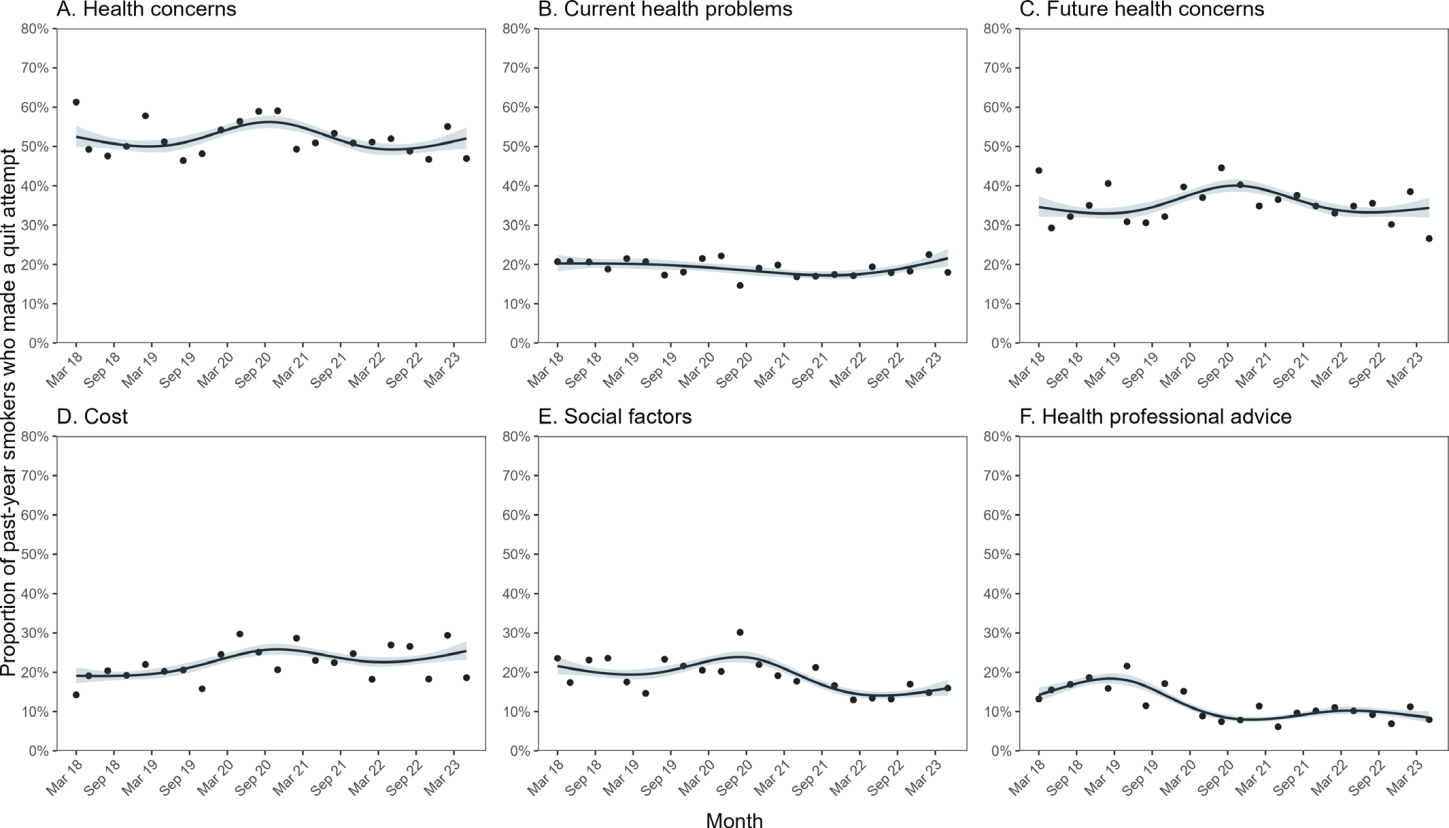
Time trends in motives for attempts to stop smoking, March 2018 to May 2023. Panels show the proportion of past-year smokers in England reporting that their most recent attempt to quit was motivated by (**A**) health concerns (current health problems, future health concerns, the coronavirus outbreak or pregnancy), (**B**) current health problems, (**C**) future health concerns, (**D**) cost, (**E**) social factors and (**F**) health professional advice. Lines represent modelled weighted prevalence by survey month, modelled non-linearly using restricted cubic splines (five knots). Shaded bands represent standard errors. Points represent observed quarterly weighted prevalence.

Figure 1 shows modelled monthly trends in motives to stop smoking and table 3 compares modelled estimates from March 2018 and May 2023 (the first and last months in the time series). Up to the start of 2020, one in two attempts to quit were motivated by health concerns (mean modelled monthly proportion, March 2018 to December 2019 = 51.0%); one in five by current health problems (20.1%) and one in three by concern about future health (33.8%). One in five was motivated by social factors (20.2%) and cost (19.9%) and one in six by health professional advice (16.5%).

**Table 3:** Modelled weighted estimates of the proportion of attempts to quit smoking motivated by health concerns, cost, social factors and health professional advice in March 2018 and May 2023

Motive	Prevalence, %*		Prevalence ratio (95% CI)†
	March 2018	May 2023	
Health concerns	52.5	52.0	0.99 (0.86 to 1.14)
Current health	20.2	21.6	1.06 (0.80 to 1.41)
Future health	34.6	34.4	0.99 (0.82 to 1.21)
Cost	19.1	25.4	1.33 (1.01 to 1.76)
Social factors	21.6	16.0	0.74 (0.54 to 1.01)
Health professional advice	14.2	8.5	0.60 (0.40 to 0.89)

* Data for March 2018 and May 2023 are weighted estimates of prevalence in these months (the first and last in the study period) from log-binomial regression with survey month modelled non-linearly using restricted cubic splines (five knots).

† Prevalence ratio for the change in prevalence across the whole time- series (May- 2023 vs March- 2018) with 95% confidence intervals calculated using bootstrapping.

CI, confidence interval

There was little overall change in the proportion of attempts to quit that were motivated by health concerns from the start to the end of the study period (PR=0.99, 95% CI 0.86 to 1.14; table 3). However, there were changes over time within this period: the proportion reporting health concerns as a motive for their most recent attempt to quit increased during 2020, peaking at 56.2% in October 2020, then returning to baseline by the end of 2021 (figure 1A). This pattern was largely driven by future health concerns (figure 1C), with negligible change over time in attempts to quit motivated by current health problems (figure 1B). Time trends differed significantly by age (interaction p=0.013; online supplemental figure 1A), social grade (interaction p=0.023; online supplemental figure 1C) and the presence of children in the household (interaction p=0.002; online supplemental figure 1D). Those aged ≥65 years and those from the most disadvantaged social grade (E) showed (i) a more pronounced rise in 2020 than those who were younger and from more advantaged social grades; and (ii) evidence of a further increase since early 2022 that was not observed among younger and more advantaged groups. Those without children in the household reported being motivated by health concerns more frequently than those with children in the household at the start of the study period, but the gap narrowed during 2020.

The proportion of attempts to quit that were motivated by cost increased significantly over the study period, from 19.1% in March 2018 to 25.4% in May 2023 (PR=1.33, 95% CI 1.01 to 1.76; table 3). This rise was non-linear: the proportion of attempts to quit motivated by cost changed very little up to 2020, increased during 2020 (peaking at 25.8% in November 2020), declined slightly during 2021 and subsequently increased from early 2022 (figure 1D). Time trends differed significantly by gender (interaction p=0.035), with cost-motivated attempts to quit increasing between 2018 and 2019 among men but declining among women, and increasing from early 2022 among women but not men (online supplemental figure 2B).

There was an uncertain decline in the proportion of attempts to quit that were motivated by social factors, from 21.6% in March 2018 to 16.0% in May 2023 (PR=0.74, 95% CI 0.54 to 1.01; table 3). Again, the trend was non-linear, with an increase during 2020 (peaking at 23.9% in August 2020) and subsequent decline during 2021 (figure 1E). There was a significant interaction with social grade (p<0.001), with the proportion of attempts to quit motivated by social factors (i) increasing between 2018 and 2019 among the most advantaged groups (AB and C1) but falling among less advantaged groups (C2 and D); (ii) showing a rise during 2020 only among the mid-range social grades (C1, C2 and D); and (iii) changing very little over the study period among the most disadvantaged group (E; online supplemental figure 3C). The rise during 2020 also appeared to be more pronounced among men online supplemental figure 3B) and those with children in the household (online supplemental figure 3D), but interactions were not statistically significant (p=0.053 and p=0.055, respectively).

The proportion of attempts to quit that were motivated by health professional advice declined significantly over the study period, from 14.2% in March 2018 to 8.5% in May 2023 (PR=0.60, 95% CI 0.40 to 0.89; table 3). It increased early in the study period, peaking at 18.4% in February 2019, then fell to 8.0% by the end of 2020, remaining relatively stable (between 8.0% and 10.3%) thereafter (figure 1F). Time trends differed significantly by gender (interaction p=0.015) and social grade (interaction p=0.012; online supplemental figure 4C). The early increase in the proportion of attempts to quit motivated by health professional advice was observed only among men (online supplemental figure 4B) and those from social grades C2 and D (the second and third least advantaged groups; online supplemental figure 4C). The subsequent fall during 2019 and 2020 appeared absent among the most and least advantaged social grades (AB and E; online supplemental figure 4C).

## Discussion

Adults in England try to stop smoking for a variety of reasons. In line with previous studies,^1^ we found that health concerns were consistently the most common factor motivating people to try to quit. The cost of smoking, social factors such as pressure from family and friends and advice from a GP or other health professional were also frequently reported. However, we observed changes in the relative contribution of these different motives to attempts to quit over recent years. Up to 2020, one in two attempts to quit were motivated by health concerns (one in three by concern about future health and one in five by current health problems), one in five by social factors and by cost and one in six by health professional advice. In 2020, the proportion of attempts to quit motivated by health concerns (driven by concern about future health), social factors and cost increased—to highs of 56.2%, 23.9% and 25.8%, respectively—and the proportion motivated by health professional advice fell to 8.0%. Rises in health-related and social motives were short-lived: the former soon returned to baseline levels and the latter fell below baseline. However, the proportion of attempts to quit motivated by cost increased further during 2022–2023 (reaching 25.4% in May 2023) and the proportion motivated by health professional advice remained suppressed.

Many of these changes are likely to have been driven by the COVID-19 pandemic, which began to affect England in March 2020. The timing of the onset of the pandemic coincided with rises in the proportion reporting health concerns (driven by concern for future health), social factors and cost as motives for trying to stop smoking. It is likely the pandemic made health concerns (an already prevalent motive) even more salient, particularly during its first year when the virus was spreading rapidly and vaccinations were not yet available. Consistent with this, we saw a more pronounced rise in attempts to quit motivated by health concerns among the oldest age group (≥65 years), who had the highest risk of mortality from COVID-19,^8^ and among those from the most disadvantaged occupational social grade, who were more likely to have pre-existing comorbidities (eg, diabetes^26^) linked to poor COVID-19 outcomes.^27^ Once the immediate threat of the virus had been attenuated via the vaccination programme, the proportion of health-related attempts to quit returned to pre-pandemic levels.

Concerns about the health risks of COVID-19 might also have led to the short-term rise in the prominence of social factors in motivating attempts to quit, if they prompted people to encourage their loved ones to try to stop smoking or if people were motivated to try to quit by others around them doing the same.^7^ The rise in attempts to quit motivated by social factors was more pronounced among mid-range social grades, who were less able to work remotely (and thus avoid exposure to infection), which might have caused concern for family and friends during the early stages of the pandemic.

Beyond its immediate risk to health, the pandemic also had wider implications. Social distancing, self-isolation and travel restrictions resulted in a reduced workforce across sectors, causing loss of income and jobs for many people.^28^ These economic pressures probably contributed to the rise in cost-motivated attempts to quit around this time. But while the pandemic’s acute risks to health—and, as a result, attempts to quit motivated by concern for health or social factors—waned over time, its economic impacts have been compounded by a cost-of-living crisis.^4^ This crisis has seen household budgets stretched as the cost of essentials has risen rapidly since late 2021, which coincides with the timing of the further, post-pandemic rise in cost-motivated attempts to quit that we observed.

Additionally, the pandemic had a substantial impact on healthcare delivery: general practice shifted to a remote delivery model to mitigate the risk of infection, lack of capacity in secondary care increased demand on GPs,^29^ and acute and urgent care were prioritised over prevention and chronic disease management.^30^ There was also evidence of patients delaying presentation to healthcare services.^31^ These circumstances might have contributed to the decline in 2020 in the proportion of attempts to quit that were motivated by health professional advice, which remained suppressed through to 2023. Healthcare services continued to be pushed to their limits as they attempted to tackle long waiting lists that built up before and during the early stages of the pandemic, while continuing to provide care to the high numbers of patients with COVID-19.^29^ This resulted in long delays to accessing healthcare, which a report by the House of Lords Public Services Committee in January 2023 described as ‘a national emergency’. In the context of this ongoing healthcare crisis, the proportion of attempts to quit motivated by health professional advice has remained low—in addition to delays, health professionals might have less time or feel less able to deliver advice remotely—and the proportion motivated by health concerns has increased among the oldest and most socioeconomically disadvantaged groups (who tend to have poorer health and therefore likely to be most affected by difficulty in accessing treatment).

These findings have implications for smoking cessation interventions and clinical practice. First, they indicate that cost is an increasingly important factor motivating people to try to stop smoking. Communicating the potential savings people can make by stopping smoking (even if they switch to alternative nicotine products^32 33^) could therefore be an effective means for motivating attempts to quit. Second, they highlight a decline in attempts to quit motivated by health professional advice. It is not clear whether this is the result of missed opportunity (ie, health professionals having reduced contact with smokers) or reduced motivation or capability (ie, health professionals not feeling motivated or able to offer advice and support for smoking cessation when seeing patients). Given attempts to quit motivated by health professional advice have remained low since the start of the pandemic while changes in those motivated by health concerns and social factors have rebounded, it seems likely to be related to the wider issues the NHS is facing rather than only the direct impacts of the pandemic. Whatever the cause may be, it is noteworthy in the context of previous research showing that attempts motivated by health professional advice are more likely to involve the use of evidence-based treatments.^2^

### Strengths and limitations

Strengths of this study include the large, representative sample and monthly data collection, permitting detailed examination of trends over time. There were also limitations. All data were self-reported, introducing scope for bias. Outcomes relied on recall of the most recent past-year attempt to quit. It is possible that participants (particularly those whose attempt to quit started longer ago) reported only the main motive that contributed to their attempt to quit and forgot other, less salient, motives. The mode of data collection changed from face-to-face to telephone interviews in April 2020. While this could have contributed to the changes in motives we observed around this time, the fact that the majority of these changes were short-lived and returned to baseline levels despite no subsequent change in methodology suggests that they are more likely to have been caused by other factors (eg, the COVID-19 pandemic). In addition, while participants were drawn from a representative sample of adults in England, results might not apply to other countries with different attitudes to smoking, tobacco control climates or provision of cessation support. Finally, we reported descriptive data on all motives captured by the survey, but we only conducted trend analyses for the four most prominent motives. There might also have been changes in other, less commonly reported motives over the study period. We also did not explore differences in motives between attempts to quit that were and were not successful, which might be an interesting direction for future research.

## Conclusion

Health concerns remain the most common motive for trying to stop smoking. The relative importance of other motives has shifted since 2020, with cost motivating a greater proportion of attempts to quit and social factors and health professional advice motivating a smaller proportion.

## supplementary material

10.1136/bmjph-2023-000420 online supplemental file 1

## Data Availability

Data are available upon reasonable request.
