# Supplementary material for: Trends in motives for trying to stop smoking: a population study in England, 2018–2023
Source: BMJ Public Health. 2024 Mar 22;2(1):e000420. doi: 10.1136/bmjph-2023-000420 (PMC11812782; doi:10.1136/bmjph-2023-000420)
Supplement: online supplemental file 1 [file bmjph-2-1-s001.pdf]

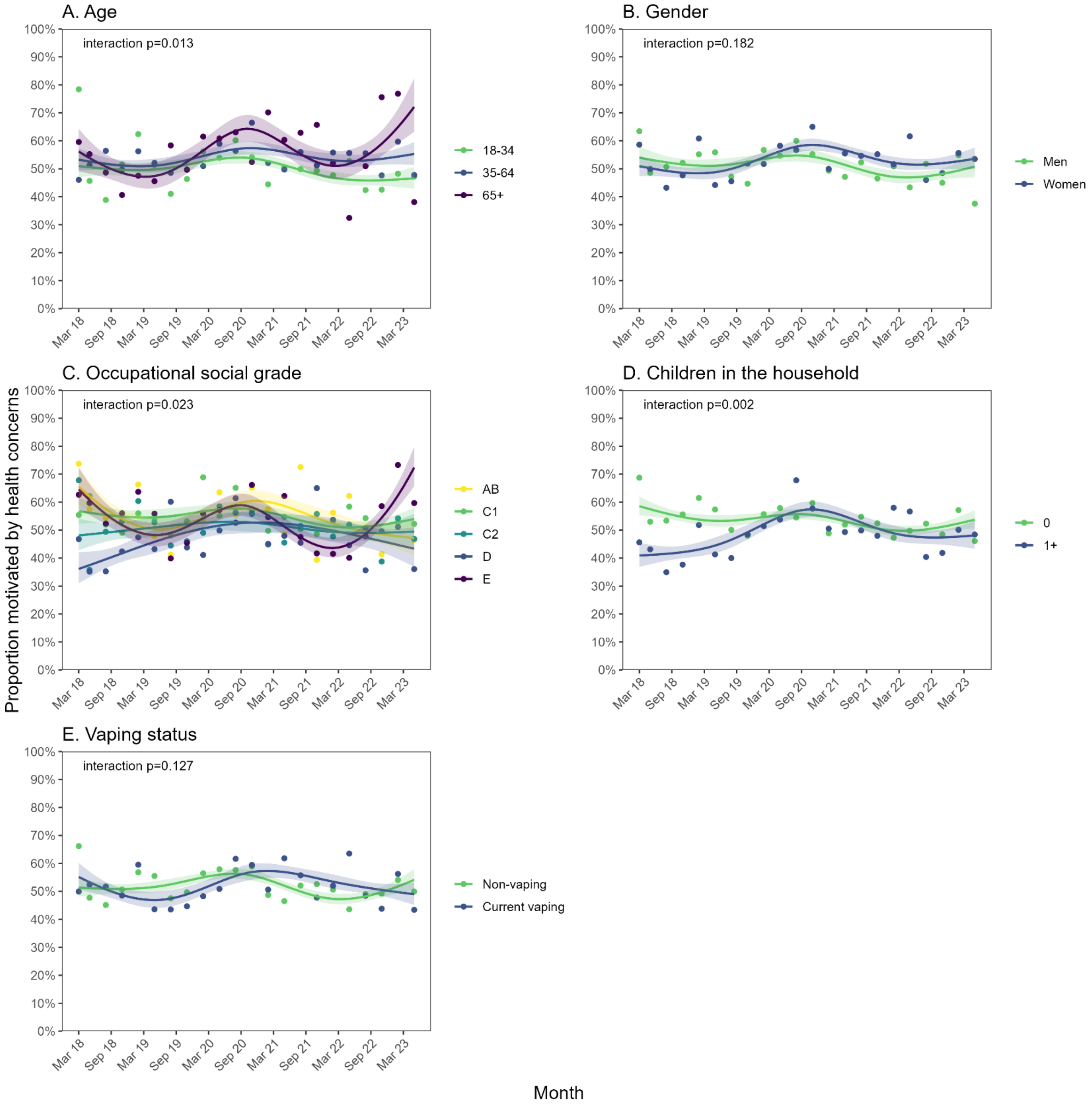

S1 Figure. Time trends in the proportion of quit attempts motivated by health concerns by (A) age, (B) gender, (C) occupational social grade, (D) children in the household, and (E) vaping status, March 2018 to May 2023. Lines represent modelled weighted prevalence by survey month, modelled non-linearly using restricted cubic splines (five knots). Shaded bands represent standard errors. Points represent observed quarterly weighted prevalence.

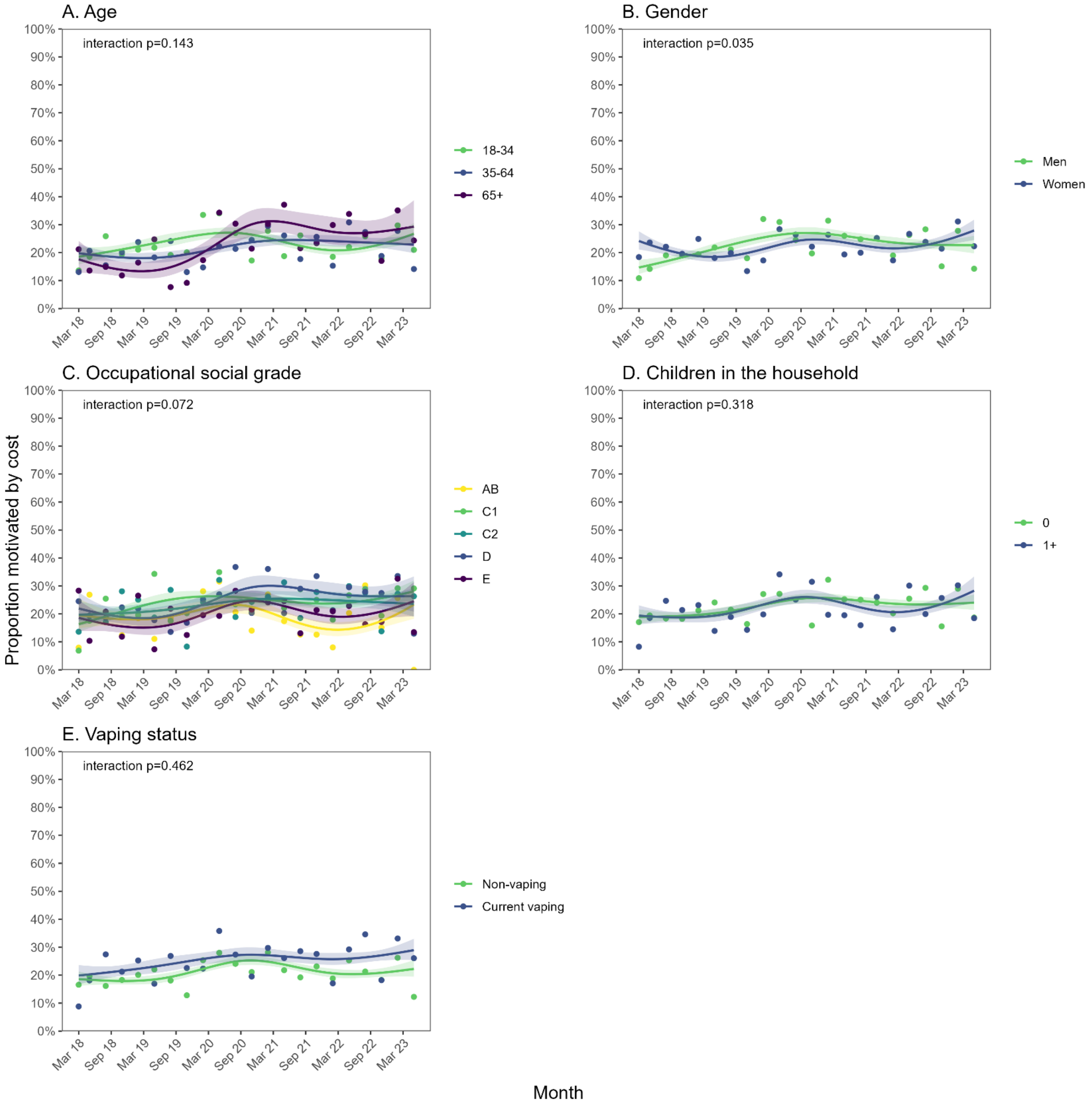

S2 Figure. Time trends in the proportion of quit attempts motivated by cost by (A) age, (B) gender, (C) occupational social grade, (D) children in the household, and (E) vaping status, March 2018 to May 2023. Lines represent modelled weighted prevalence by survey month, modelled non-linearly using restricted cubic splines (five knots). Shaded bands represent standard errors. Points represent observed quarterly weighted prevalence.

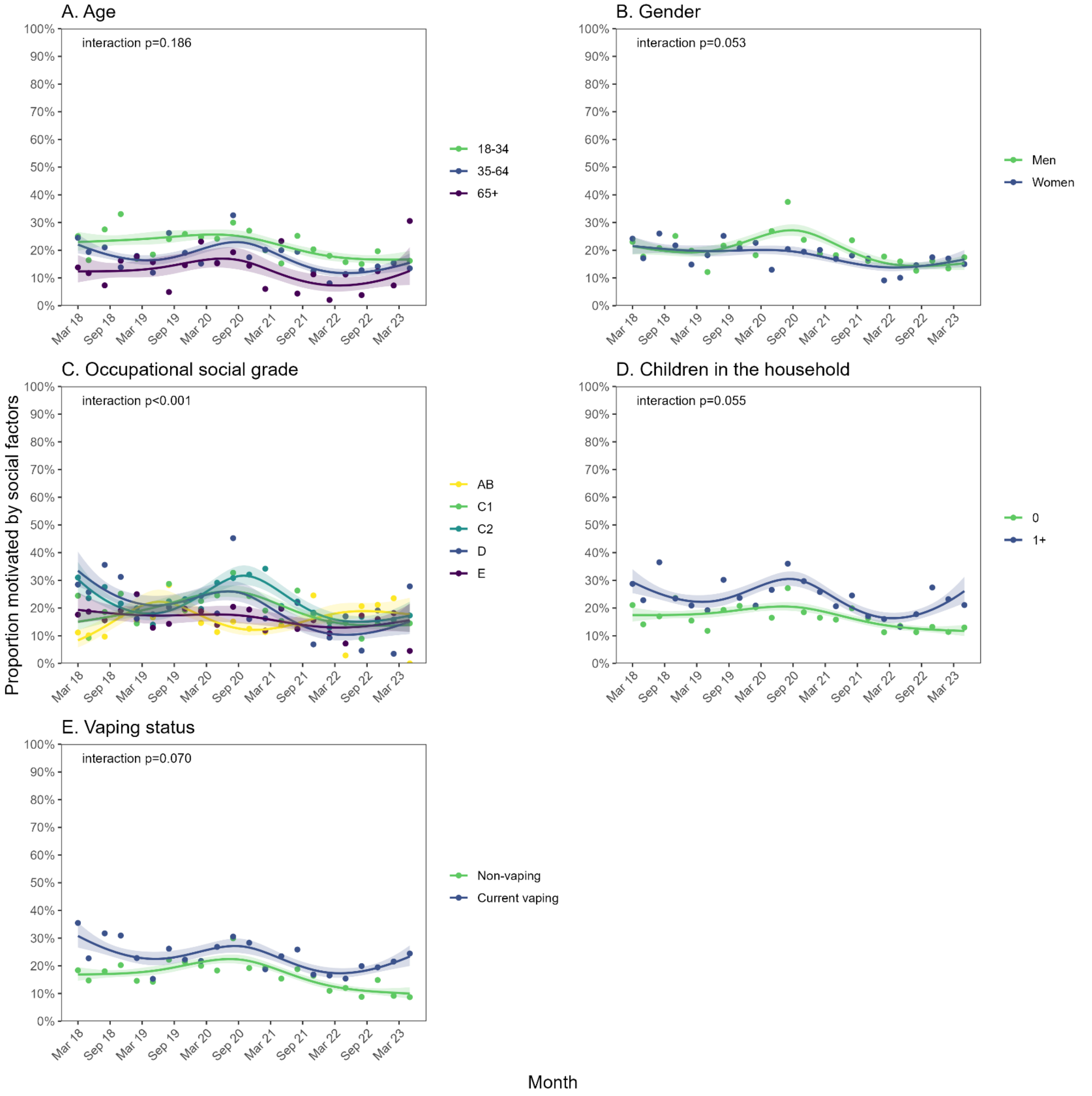

S3 Figure. Time trends in the proportion of quit attempts motivated by social factors by (A) age, (B) gender, (C) occupational social grade, (D) children in the household, and (E) vaping status, March 2018 to May 2023. Lines represent modelled weighted prevalence by survey month, modelled non-linearly using restricted cubic splines (five knots). Shaded bands represent standard errors. Points represent observed quarterly weighted prevalence.

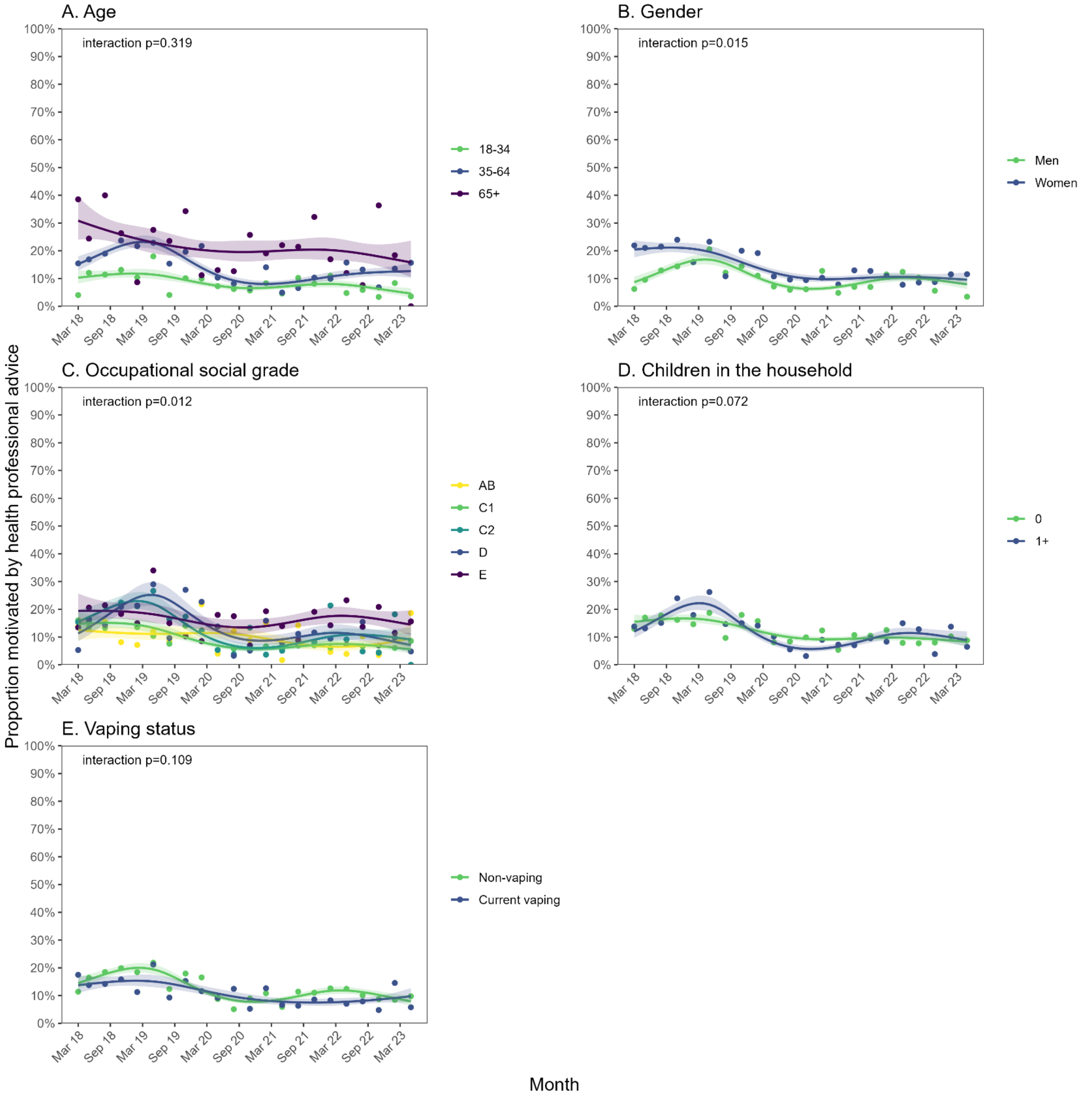

S4 Figure. Time trends in the proportion of quit attempts motivated by health professional advice by (A) age, (B) gender, (C) occupational social grade, (D) children in the household, and (E) vaping status, March 2018 to May 2023. Lines represent modelled weighted prevalence by survey month, modelled non-linearly using restricted cubic splines (five knots). Shaded bands represent standard errors. Points represent observed quarterly weighted prevalence.
